# One-Pot Synthesis of Biochar from Industrial Alkali Lignin with Superior Pb(II) Immobilization Capability

**DOI:** 10.3390/molecules29184310

**Published:** 2024-09-11

**Authors:** Jiale Li, Taoze Liu, Zhanghong Wang

**Affiliations:** 1 College of Eco-Environmental Engineering, Guizhou Minzu University, Guiyang 550025, China; 15885794651@163.com; 2 Engineering Research Center of Green and Low-Carbon Technology for Plastic Application, Guizhou Minzu University, Guiyang 550025, China; 3 Key Laboratory of Energy Thermal Conversion and Control of Ministry of Education, School of Energy and Environment, Southeast University, Nanjing 210096, China

**Keywords:** industrial alkali lignin, pyrolysis, biochar, adsorption, Pb(II)

## Abstract

This study synthesized biochar through a one-pot pyrolysis process using IALG as the raw material. The physicochemical properties of the resulting biochar (IALG-BC) were characterized and compared with those of biochar derived from acid-treated lignin with the ash component removed (A-IALG-BC). This study further investigated the adsorption performances and mechanisms of these two lignin-based biochars for Pb(II). The results revealed that the high ash content in IALG, primarily composed of Na, acts as an effective catalyst during pyrolysis, reducing the activation energy and promoting the development of the pore structure in the resulting biochar (IALG-BC). Moreover, after pyrolysis, Na-related minerals transformed into particulate matter sized between 80 and 150 nm, which served as active adsorption sites for the efficient immobilization of Pb(II). Adsorption results demonstrated that IALG-BC exhibited a significantly superior adsorption performance for Pb(II) compared to that of A-IALG-BC. The theoretical maximum adsorption capacity of IALG-BC for Pb(II), derived from the Langmuir model, was determined to be 809.09 mg/g, approximately 40 times that of A-IALG-BC. Additionally, the adsorption equilibrium for Pb(II) with IALG-BC was reached within approximately 0.5 h, whereas A-IALG-BC required more than 2 h. These findings demonstrate that the presence of inorganic mineral components in IALG plays a crucial role in its resource utilization.

## 1. Introduction

Lignin, as the second largest natural polymer in nature after cellulose, has a high carbon content (30–40 wt.%), abundant surface functional groups, and resistance to biochemical degradation, making it widely applicable in many fields [1]. However, lignin is a by-product of the papermaking industry, with an annual global production exceeding 50 million tons, leading to severe environmental issues [2,3]. Currently, lignin is mainly used as a low-value fuel for power generation, with only about 2% being developed into high-value functional materials such as water reducers, adhesives, rubber, and carbon fibers [1]. The resource utilization of lignin is primarily constrained by its unique physical and chemical properties, such as its richness in organic and inorganic impurities and complex structure [4]. The development of green, efficient technologies for the resource utilization of lignin is urgent and of significant importance.

Accompanied by the development of socio-economic conditions, the extensive use of heavy metal products has brought about severe heavy metal pollution [5]. These pollutants can gradually accumulate and intensify through the food chain, ultimately posing a threat to human health [6]. For example, the intake of lead can adversely affect the human brain and nervous system, as well as damage the hematopoietic system and cause irreversible harm to internal organs such as the kidneys and liver [7,8]. Consequently, there has been widespread attention paid towards developing targeted technologies to address heavy metal pollution [9]. Currently, the primary methods for treating heavy metal-containing wastewater include chemical precipitation, electrochemical treatment, ion exchange, membrane filtration, phytoremediation, and adsorption [10,11,12]. Among these, adsorption is widely promoted due to its simplicity, high removal efficiency, strong applicability, reusability, and low cost [10,13]. Biochar, which is produced by the pyrolysis of biomass materials under limited or oxygen-free conditions, has a high carbon content, well-developed porous structure, and abundant surface functional groups [14]. Extensive research has demonstrated that biochar exhibits an excellent adsorption performance for heavy metal ions in water, making it a highly promising adsorbent [9].

Notably, biochar is constrained by its inherent surface charge, pore structure, and active adsorption sites, which result in an overall inferior heavy metal adsorption performance compared to other adsorbents like activated carbon, molecular sieves, and MOFs [10,13]. For example, research by Tan et al. found that biochar prepared from apple tree branches had a theoretical maximum adsorption capacity for Pb(II) of 53.90 mg/g [15]. In contrast, Zhang et al. reported that activated carbon derived from rape straw had a theoretical maximum adsorption capacity for Pb(II) of 253.20 mg/g, which is about five times that of apple tree branch biochar [16]. Studies have shown that functionalized biochar with metal salts can significantly enhance its heavy metal adsorption performance, garnering widespread attention [17,18]. Typically, metal salts perform a variety of functions: they serve as activators to facilitate the evolution of biochar’s pore architecture, offer plentiful adsorption sites by acting as active centers, and function as modifiers to alter the physicochemical attributes of biochar, such as the surface charge and pH [9,19]. For example, Chen et al. discovered that pomelo peel biochar modified with FeCl_3_ and Na_2_CO_3_ achieved a theoretical maximum adsorption capacity for Pb(II) of 205.39 mg/g, over two times that of pristine biochar [17]. While metal salt treatment improves the adsorption performance of biochar, it also increases production costs and poses secondary environmental issues. Interestingly, certain biomass sources, being agro-industrial by-products, are naturally rich in metal minerals and can directly produce high-performance biochar, such as industrial alkaline lignin [20].

In this study, industrial alkali lignin (IALG), rich in metal minerals, was used as a raw material to produce biochar (IALG-BC) through direct pyrolysis at 700 °C using a one-pot method. The surface chemical characteristics, pore features, surface functional groups, and surface morphology of IALG-BC were analyzed, and its effectiveness in immobilizing Pb(II) in wastewater was examined. Additionally, biochar derived from acid-treated IALG, which had its ash content removed (acid-treated IALG-based biochar (A-IALG-BC)), was used as a comparison to elucidate the role of ash (metal minerals) in the preparation and application process of biochar. This research aims to provide new perspectives and technical support for the resourceful utilization of industrial alkali lignin.

## 2. Results and Discussion

### 2.1. Thermal Behavior and Kinetic Analysis of Lignin

**Figure 1:**
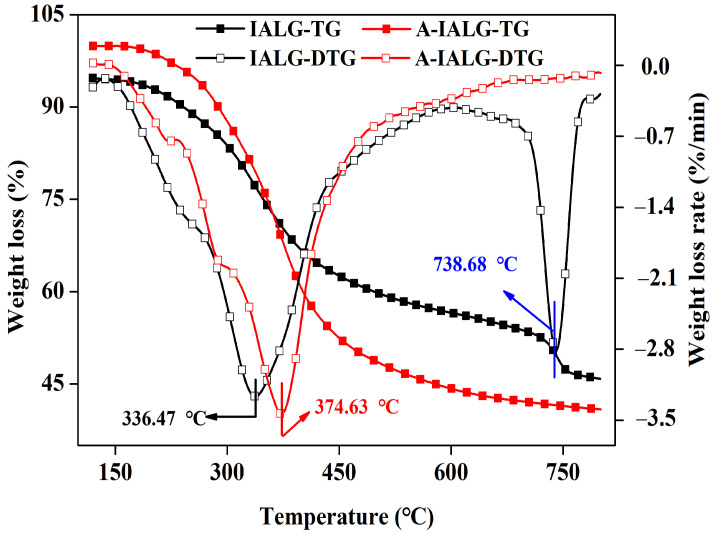
Thermal pyrolysis behavior of IALG and A-IALG.

Thermogravimetric analysis (TGA) was utilized to investigate the pyrolysis behavior of IALG and A-IALG. As illustrated in Figure 1 (TG and DTG curves), the pyrolysis process of IALG primarily encompasses three stages: the dehydration stage (room temperature to 153.65 °C), the main pyrolysis stage of lignin (153.65 to 518.45 °C), and the carbonization stage (518.45 to 800 °C). The precursor of IALG, specifically the lignin in the biomass, possesses a high oxygen content (18–40 wt.%) and an abundance of oxygen-containing functional groups, which confer relatively good water retention properties onto IALG. As the pyrolysis temperature escalates, the free water within IALG evaporates, leading to a mass loss in IALG. With a continued increase in temperature, the lignin undergoes considerable decomposition. Nevertheless, due to the intricate chemical structure of lignin, which comprises both less thermally stable side chains and more thermally stable chemical bonds, the pyrolysis range of lignin is extensive, resulting in a broad and blunt peak on the DTG curve [21]. The most significant mass loss occurs during the primary pyrolysis stage of lignin (up to 35.33 wt.%), with the maximum weight loss rate and maximum constant rate occurring at −3.26 wt.%/min and 336.47 °C, respectively. Following the termination of lignin pyrolysis, the reaction proceeds to the carbonization stage. In this stage, the carbon structure derived from lignin undergoes further decomposition, and the pore structure progressively forms, resulting in relatively minor mass loss. Notably, a distinct narrow weight loss peak centered at 738.68 °C is observed for IALG during this stage, likely attributable to the decomposition of the minerals present in IALG. Comparatively, the TG and DTG curves of A-IALG exhibit a similar profile to that of IALG, with the exception of the weight loss stage near 738 °C. This observation further corroborates that the weight loss peak in IALG is due to the mineral content, which is largely removed in A-IALG post-acid treatment. Moreover, the initial pyrolysis temperature and the temperature corresponding to the maximum pyrolysis rate of A-IALG during the main pyrolysis stage are 163.53 °C and 374.63 °C, respectively, both of which are significantly higher than those of IALG. This indicates that the mineral content in IALG facilitates self-catalysis, lowering the pyrolysis temperature and promoting the decomposition of IALG. Similar findings can be found in previous studies [22,23].

Kinetic parameters, including the activation energy (*E*) and the pre-exponential factor (*A*), can be used to qualitatively characterize the pyrolysis behavior of reactants. These parameters can be derived from thermogravimetric analysis (TGA) data. To further substantiate the catalytic influence of minerals within IALG on its pyrolysis process, a first-order kinetic model was employed to fit the primary pyrolysis processes of IALG and A-IALG, and the corresponding pyrolysis parameters were calculated according to Equations (1)–(3) [24]. As presented in Table 1, the *E* of IALG was determined to be 14.27 J/mol, approximately one-third of that of A-IALG. This indicates that the energy required for the reaction during the primary pyrolysis stage of IALG is significantly lower. This observation aligns with the results showing that both the initial pyrolysis temperature and the temperature corresponding to the maximum pyrolysis rate during the primary pyrolysis stage of IALG were lower than those of A-IALG. These findings suggest that the higher mineral content in IALG can effectively reduce the energy demand during its reaction process, thereby facilitating its extensive decomposition. This conclusion is consistent with the results reported in previous studies [25].

(1) $$\frac{dx}{dt}=A\exp\left[\left(\frac{-E}{RT}\right)\left(1-x\right)\right]$$

(2) $$x=\frac{w_{0}-w_{t}}{w_{0}-w_{f}}$$

(3) $$\ln\left[\frac{-\ln\left(1-x\right)}{T^{2}}]=\ln[\frac{AR}{HE}\left(1-\frac{2RT}{E}\right)\right]-\frac{E}{RT}$$

where *E* is the activation energy (kJ/mol); *A* is the pre-exponential factor (1/min); *T* is the temperature (K); *t* is the time (min); *R* is a universal gas constant (J/mol K); *x* is the conversion of feedstock; *w*_0_ is the initial mass of the sample (mg); *w*_t_ is the mass of the sample at time (t); and *w*_f_ is the mass of the sample at the end of pyrolysis.

### 2.2. Physicochemical Properties of Biochar

#### 2.2.1. Porosity Analysis

**Figure 2:**
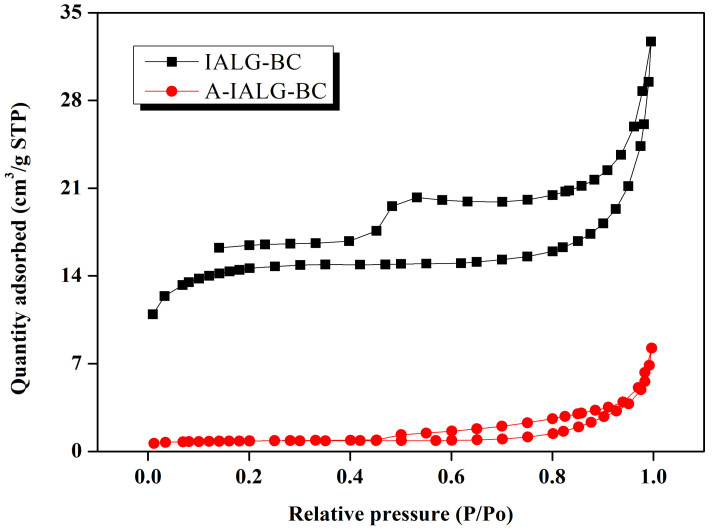
N_2_ adsorption/desorption isotherm curves of IALG-BC and A-IALG-BC.

The N_2_ adsorption/desorption isotherms of IALG-BC and A-IALG-BC are depicted in Figure 2. According to the International Union of Pure and Applied Chemistry (IUPAC) classification, the N_2_ adsorption/desorption isotherm of IALG-BC corresponds to a hybrid of type I and type IV, with an H4 type hysteresis loop [26]. This indicates that IALG-BC features a rich pore structure, predominantly composed of micropores and mesopores. In contrast, the N_2_ adsorption/desorption isotherm of A-IALG-BC can be classified as a hybrid of type III and type IV, with an H3 type hysteresis loop. This pattern suggests that A-IALG-BC is characterized by the dominant presence of macropores and an uneven pore structure.

The pore analysis results, as depicted in Table 2, demonstrate that the specific surface area of IALG-BC is 106.65 m^2^/g, which is approximately 50 times greater than that of A-IALG-BC, measured at 2.81 m^2^/g. This significant disparity indicates that IALG-BC exhibits a markedly more developed pore structure. The enhanced porosity of IALG-BC can be attributed to the abundant minerals present in lignin. Previous studies have documented that alkali metal salts, such as NaOH, KOH, and Na_2_CO_3_, possess unique catalytic properties and pore-forming capabilities, making them effective activating agents for the synthesis of porous carbon materials [27,28]. IALG inherently contains a substantial amount of Na-related minerals (see Section 3), which likely facilitate the formation and growth of the pore structure in the resulting carbon materials based on activation mechanisms such as those depicted in Equations (4)–(8):

(4) $$6\mathrm{NaOH}+2C\to 2\mathrm{Na}+3H_{2}+2{\mathrm{Na}}_{2}{\mathrm{CO}}_{3}$$

(5) $${\mathrm{Na}}_{2}{\mathrm{CO}}_{3}\to {\mathrm{Na}}_{2}O+{\mathrm{CO}}_{2}$$

(6) $${\mathrm{CO}}_{2}+C\to 2\mathrm{CO}$$

(7) $${\mathrm{Na}}_{2}{\mathrm{CO}}_{3}+2C\to 2\mathrm{Na}+3\mathrm{CO}$$

(8) $$C+{\mathrm{Na}}_{2}O\to 2\mathrm{Na}+\mathrm{CO}$$

#### 2.2.2. Surface Functional Group Analysis

**Figure 3:**
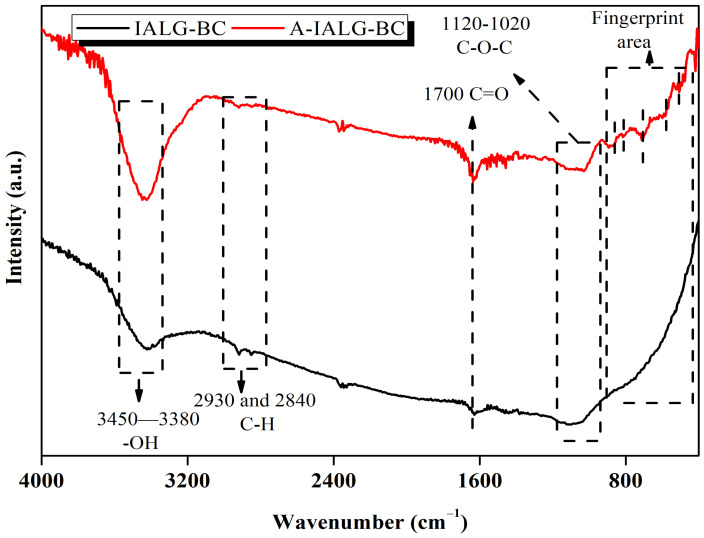
Surface functional groups of IALG-BC and A-IALG-BC.

The FT-IR spectra of IALG-BC and A-IALG-BC, as illustrated in Figure 3, indicate a diverse array of surface functional groups. Notably, the absorption peak in the range of 3450–3380 cm^−1^ is attributed to the stretching vibrations of -OH groups [29]. The absorption peaks observed at 2930 and 2840 cm^−1^ correspond to the symmetric and asymmetric stretching vibrations of C-H bonds [29]. The peak at 1700 cm^−1^ is indicative of the stretching vibrations of C=O groups, while the absorption peaks located between 1120 and 1020 cm^−1^ are associated with the stretching vibrations of C-O-C groups [30,31]. In contrast, the FT-IR spectrum of A-IALG-BC reveals surface functional groups similar to those of IALG-BC, but with generally more intense peaks. This suggests that A-IALG-BC possesses a greater abundance of surface functional groups, particularly oxygen-containing functionalities such as -OH, C=O, and C-O-C groups. Consistent with prior findings, it can be inferred that the presence of minerals in IALG facilitates deoxygenation reactions during the pyrolysis process [32]. Additionally, the fingerprint region of A-IALG-BC exhibits numerous absorption peaks (860, 812, 705, 585, and 510 cm^−1^), primarily attributed to the stretching and bending vibrations of C-H bonds within the carbon skeletal structure and the vibrations of other single-bond surface functional groups [33,34]. These functional groups are notably absent in IALG-BC, indicating that the substantial mineral content in IALG not only aids in deoxygenation but also promotes further dehydrogenation in the resulting carbon material.

#### 2.2.3. Surface Morphology Analysis

**Figure 4:**
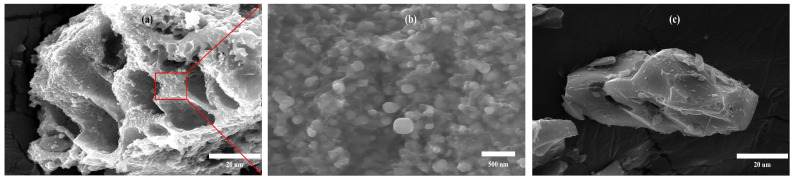
SEM images of biochars: (**a**) 5000× IALG-BC; (**b**) 150,000× IALG-BC; (**c**) 5000× A-IALG-BC.

The SEM images of IALG-BC and A-IALG-BC, as depicted in Figure 4, reveal distinct morphological differences. The surface of IALG-BC is characterized by its rough texture and an extensive, well-developed pore structure, corroborating the findings from the N_2_ adsorption/desorption isotherm analysis. Closer inspection indicates that the pore framework of IALG-BC is interspersed with numerous particulate matter, with diameters ranging from 80 to 150 nm. Given the relatively high ash content of the precursor material, it is inferred that these particles predominantly consist of Na-related minerals [35,36]. The distinctive pore architecture of IALG-BC, marked by its rough surface and abundant mineral particulates, imparts a high capacity for the adsorption and immobilization of pollutants. In contrast, the surface morphology of A-IALG-BC appears relatively smooth and lacks a discernible pore structure. This observation can be attributed to the acid treatment process, which effectively removed a substantial portion of the ash content from the raw material. Consequently, the processed material exhibits a less developed pore network compared to IALG-BC.

#### 2.2.4. Crystalline Mineral Analysis

**Figure 5:**
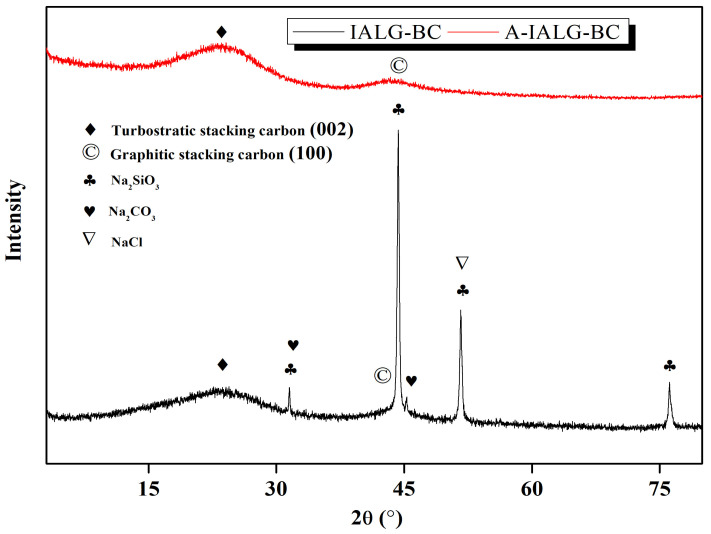
XRD patterns of IALG-BC and A-IALG-BC.

The crystalline phases of the minerals present in IALG-BC and A-IALG-BC were investigated using X-ray diffraction (XRD) analysis, with the results depicted in Figure 5. The XRD patterns reveal that IALG-BC contains a substantial amount of Na-related minerals, specifically Na_2_SiO_3_, Na_2_CO_3_, and NaCl, underscoring the significance of Na as a mineral constituent in IALG-BC [36]. Complementary to the SEM observations, it is apparent that these Na-related minerals predominantly exist as nanoparticles distributed on the surface of IALG-BC. Conversely, A-IALG-BC does not exhibit any prominent mineral diffraction peaks, which is consistent with the removal of ash during the acid treatment process. This observation aligns with the SEM findings presented in Figure 4. Moreover, the XRD pattern of A-IALG-BC displays two broad diffraction peaks around 23° and 45°, corresponding to the (002) reflection and the superposition of (100) reflections of the graphitic-type lattice, respectively [37].

### 2.3. Adsorption Performance of Industrial Alkali Lignin-Based Biochars

#### 2.3.1. Effect of Pb(II) Initial Concentration

**Figure 6:**
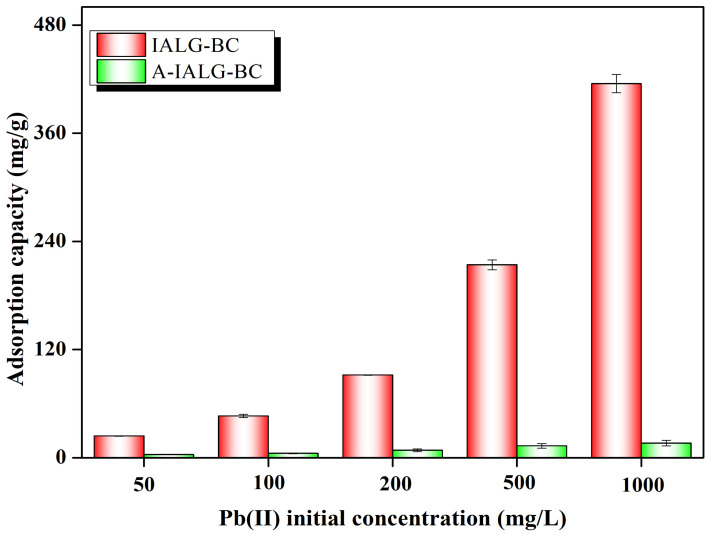
Effect of Pb(II) initial concentration of the adsorption capabilities of IALG-BC and A-IALG-BC.

As illustrated in Figure 6, IALG-BC exhibits an exceptional adsorption performance for Pb(II), with the corresponding adsorption capacity progressively increasing with the initial concentration of Pb(II). For example, at an initial Pb(II) concentration of 50 mg/L, the adsorption capacity of IALG-BC is 24.11 mg/g. This capacity escalates to a maximum of 414.98 mg/g when the initial Pb(II) concentration is elevated to 1000 mg/L. This enhanced adsorption capability can be attributed to the abundance of active adsorption sites on the surface of IALG-BC. At higher concentrations of Pb(II), the increased mass transfer driving force improves the interaction between Pb(II) ions and the adsorbent, thereby promoting the adsorption reaction [38]. In contrast, while the initial Pb(II) concentration similarly influences A-IALG-BC, its adsorption capacities are markedly lower, ranging from 3.30 to 16.18 mg/g. This significant difference further underscores the role of Na-related minerals in IALG-BC, which act as highly efficient active adsorption sites for Pb(II), thereby substantially enhancing its adsorption capacity.

#### 2.3.2. Effect of Contact Time

**Figure 7:**
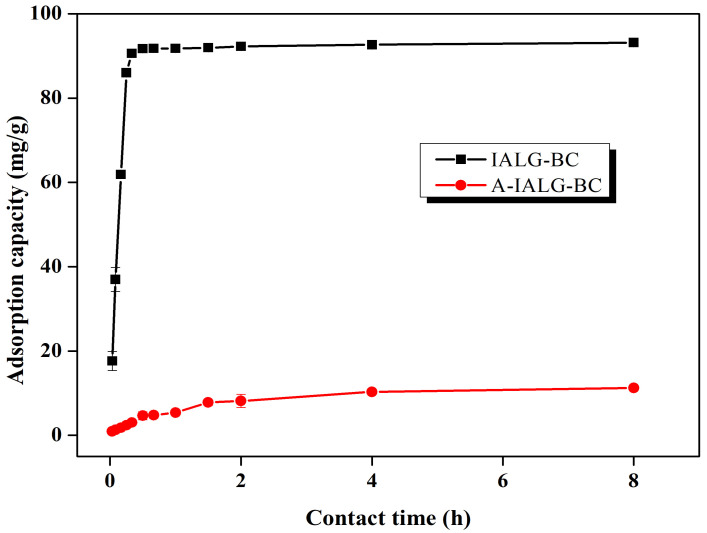
Effect of contact time on the adsorption capabilities of IALG-BC and A-IALG-BC.

The adsorption capacities of IALG-BC and A-IALG-BC for Pb(II) as a function of the reaction time are illustrated in Figure 7. IALG-BC exhibits rapid Pb(II) adsorption within the initial 0.5 h, during which the adsorption capacity increases sharply, accounting for 98.44% of the total adsorption capacity. As the reaction time extends, the adsorption rate of Pb(II) by IALG-BC decelerates, showing only minor variations (a range of 1.46 mg/g), indicating that the system is approaching equilibrium. The short equilibrium time for Pb(II) adsorption by IALG-BC suggests that the process is predominantly governed by chemical interactions rather than physical processes such as pore filling [39]. Typically, chemical adsorption processes are faster, whereas physical adsorption processes tend to be more time-consuming [40]. Based on prior analyses, it is evident that the Na-related minerals in IALG-BC serve as active adsorption sites, primarily facilitating Pb(II) uptake through chemical mechanisms such as precipitation and ion exchange. During the initial phase of the reaction, the surface of IALG-BC possesses numerous available active adsorption sites, which accelerates the adsorption process and leads to a rapid increase in the adsorption capacity. As the reaction progresses, these active sites are gradually depleted, reducing the number of available sites and consequently slowing down the adsorption process [20]. In contrast, the adsorption capacities of A-IALG-BC at various reaction times are consistently lower than those of IALG-BC, with the adsorption process attaining equilibrium only after approximately 2 h. This indicates that the removal of ash results in a decreased adsorption capacity and a slower adsorption rate for the resulting biochar.

#### 2.3.3. Effect of Ambient Temperature

**Figure 8:**
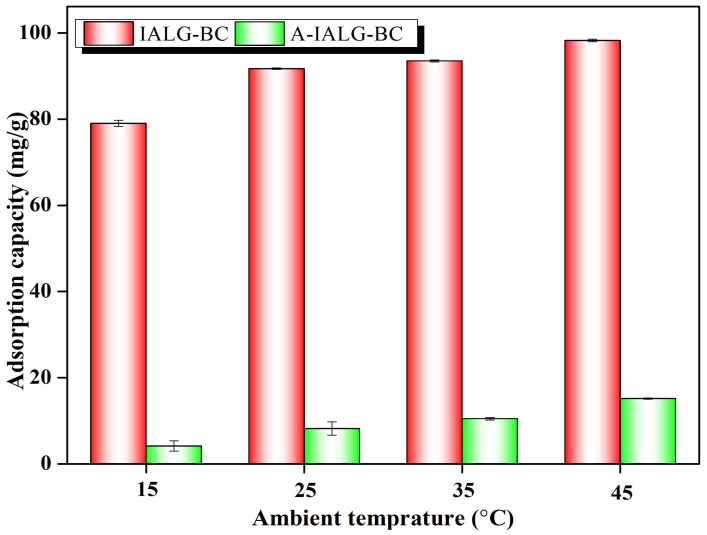
Effect of ambient temperature on the adsorption capabilities of IALG-BC and A-IALG-BC.

As depicted in Figure 8, the impacts of the ambient temperature on the adsorption of Pb(II) by both IALG-BC and A-IALG-BC exhibit a similar pattern, whereby an increase in temperature enhances the adsorption capacity of the respective biochars. For example, at an ambient temperature of 15 °C, the adsorption capacities of IALG-BC and A-IALG-BC for Pb(II) are 79.02 mg/g and 4.16 mg/g, respectively. When the temperature is elevated to 45 °C, the corresponding adsorption capacities rise to 98.27 mg/g and 15.17 mg/g, respectively. This phenomenon can be attributed to the increased thermal motion of adsorbates (such as Pb(II)) at higher temperatures, which enhances the likelihood of collisions between adsorbates and adsorption sites, particularly those that are less accessible at lower temperatures, thus facilitating the adsorption process [16]. Additionally, some adsorption reactions are endothermic and require an input of energy; thus, an increase in ambient temperature can supply the necessary energy to enhance the adsorption efficiency. It is well documented that the adsorption processes for pollutants by most biochars are endothermic and energy-consuming [16,17].

#### 2.3.4. Effect of Solution pH

**Figure 9:**
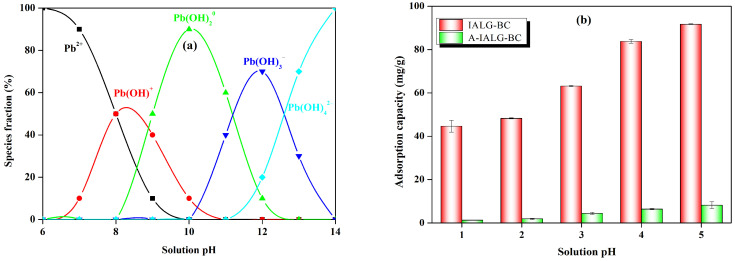
Forms of Pb(II) at different solution pH values (**a**) and effect of solution pH on the adsorption capabilities of IALG-BC and A-IALG-BC (**b**).

The pH of the solution exerts a direct influence on the speciation of the adsorbate as well as on the surface charge and other chemical properties of the adsorbent, consequently affecting the adsorption process (see Figure 9a). Accordingly, the selected pH range for investigating the impact of the solution pH on the Pb(II) adsorption by IALG-BC and A-IALG-BC was 1–5 (see Figure 9b). Generally, as the pH of the solution increases, there is a gradual enhancement in the adsorption capacities of IALG-BC and A-IALG-BC for Pb(II), indicating that higher solution pH values facilitate the adsorption reaction. At lower solution pH values (pH = 1–2), the adsorption performances of both IALG-BC and A-IALG-BC for Pb(II) are relatively poor. This can be attributed to the protonation of the biochar surface (Equations (9) and (10)) and the partial degradation of active adsorption sites (Equations (11) and (12)) under these acidic conditions. The protonated biochar surface experiences strong electrostatic repulsion with Pb(II), which impedes the contact between Pb(II) and the adsorption sites. Additionally, the high concentration of H^+^ ions in the solution compete vigorously with Pb(II) for adsorption sites. As the solution pH increases, the biochar surface undergoes deprotonation, accompanied by a decrease in the concentration of H^+^ ions in the solution. This reduction in electrostatic repulsion between the biochar surface and Pb(II) transitions into electrostatic attraction, substantially enhancing the likelihood of Pb(II) contacting the active adsorption sites [17]. Consequently, the adsorption performance of biochar for Pb(II) improves progressively with increasing solution pH values.

(9) $$\mathrm{Biochar}-\mathrm{COOH}+H^{+}\to \mathrm{Biochar}-{\mathrm{COOH}}_{2}^{+}$$

(10) $$\mathrm{Biochar}-\mathrm{OH}+H^{+}\to \mathrm{Biochar}-{\mathrm{OH}}_{2}^{+}$$

(11) $${\mathrm{Na}}_{2}{\mathrm{CO}}_{3}+2H^{+}\to H_{2}{\mathrm{CO}}_{3}+2{\mathrm{Na}}^{+}$$

(12) $${\mathrm{Na}}_{2}{\mathrm{SiO}}_{3}+2H^{+}\to H_{2}{\mathrm{SiO}}_{3}↓+2{\mathrm{Na}}^{+}$$

### 2.4. Potential Adsorption Mechanism Analysis

#### 2.4.1. Adsorption Isotherm Analysis

The distribution of the adsorbate between the solution phase and the adsorbent surface during the adsorption process was modeled using adsorption isotherm equations. Specifically, the Langmuir and Freundlich isotherm models were employed, corresponding to Equation (13) and Equation (14), respectively:

(13) $$Q_{e}=\frac{Q_{m}K_{l}C_{e}}{1+K_{l}C_{e}}$$

(14) $$Q_{e}=K_{f}C_{e}^{1/n}$$

In these equations, *Q*_e_ denotes the equilibrium adsorption capacity (mg/g); *C*_e_ represents the equilibrium concentration post-adsorption (mg/L); *Q*_m_ is the theoretical maximum adsorption capacity (mg/g); *K*_l_ is the Langmuir adsorption constant (L/mg); *K*_f_ is the Freundlich affinity coefficient (mg^(1−n)^L^n^/g); and *n* is the Freundlich heterogeneity factor.

**Figure 10:**
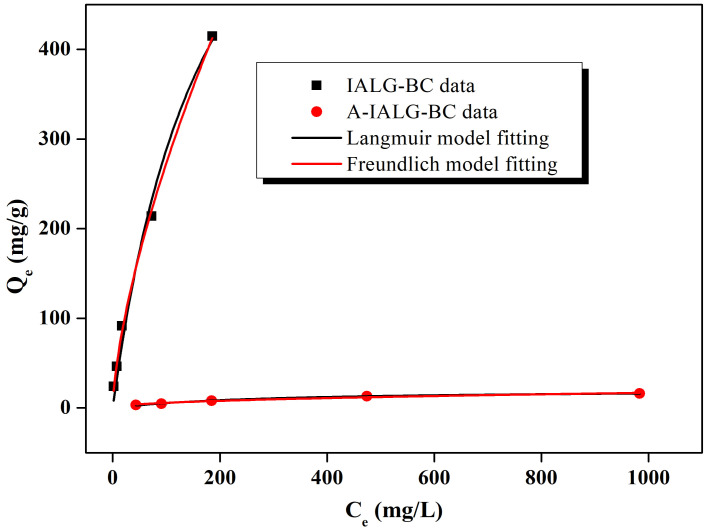
Adsorption isotherm fitting for the adsorption of Pb(II) on IALG-BC and A-IALG-BC.

The adsorption isotherm models for Pb(II) adsorption by IALG-BC and A-IALG-BC were fitted, and the results are presented in Figure 10 and Table 3. The correlation coefficients for the Langmuir model fitting of Pb(II) adsorption by IALG-BC and A-IALG-BC are 0.997 and 0.995, respectively. These values are significantly higher than those obtained from the Freundlich model (0.961–0.965), indicating that the Langmuir model provides a more accurate description of the adsorption process. This suggests that the adsorption of Pb(II) by IALG-BC and A-IALG-BC predominantly follows homogeneous monolayer adsorption. This conclusion is supported by SEM and XRD analyses (Figure 4 and Figure 5), which reveal that the Na-related active adsorption sites in IALG-BC are uniformly dispersed on its surface as nanoparticles. According to the Langmuir model, the theoretical maximum adsorption capacity of IALG-BC for Pb(II) is 809.09 mg/g, which is approximately 40 times higher than that of A-IALG-BC, highlighting the crucial role of Na-related minerals. Furthermore, the Pb(II) adsorption capacity of IALG-BC exceeds that of most biomass-based adsorbents and is comparable to some high-cost commercial adsorbents, indicating that IALG-BC has significant potential as a highly effective adsorbent (see Table 4) [41,42].

#### 2.4.2. Adsorption Kinetic Analysis

The distribution of the adsorbate on the adsorbent surface over time during the adsorption process can be effectively described using adsorption kinetics models. Specifically, the pseudo-first-order and pseudo-second-order models are represented by Equations (15) and (16), respectively:

(15) $$\frac{dQ_{t}}{dt}=k_{1}\left(Q_{e}-Q_{t}\right)$$

(16) $$\frac{dQ_{t}}{dt}=k_{2}{\left(Q_{e}-Q_{t}\right)}^{2}$$

In these equations, *t* denotes the reaction time (h); *Q*_t_ represents the adsorption capacity at time (t) (mg/g); *Q*_e_ is the equilibrium adsorption capacity (mg/g); *k*_1_ is the adsorption rate constant for the pseudo-first-order model (h^−1^); and *k*_2_ is the adsorption rate constant for the pseudo-second-order model (g/(mg·h)). 

**Figure 11:**
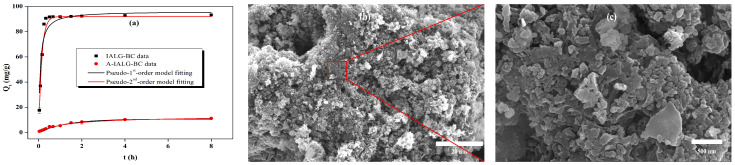
Adsorption kinetic fitting for the adsorption of Pb(II) on IALG-BC and A-IALG-BC (**a**), SEM image of 5000× IALG-BC after Pb(II) adsorption (**b**) and SEM image of 150,000× IALG-BC after Pb(II) adsorption (**c**).

As depicted in Figure 11a and detailed in Table 5, the pseudo-second-order model provides a superior fit for the adsorption process of Pb(II) by IALG-BC and A-IALG-BC, with correlation coefficients ranging from 0.989 to 0.991, significantly higher than those obtained from the pseudo-first-order model (0.906–0.924). Furthermore, the equilibrium adsorption capacities (*Q*e) derived from the pseudo-second-order model for IALG-BC and A-IALG-BC are 92.96 mg/g and 13.00 mg/g, respectively, closely aligning with the experimental equilibrium adsorption capacities (qe). These results suggest that the adsorption of Pb(II) by IALG-BC and A-IALG-BC is predominantly governed by chemisorption mechanisms [47]. As previously discussed, a substantial quantity of Na-related nanomineral particles is distributed on the surface of IALG-BC, enabling the adsorption and immobilization of Pb(II) through precipitation mechanisms (see Equations (17) and (18)) [35,48]. The SEM image of IALG-BC following Pb(II) adsorption is shown in Figure 11b. It can be observed that the nanomineral particles on the IALG-BC surface disappear, replaced by a dense aggregation of fluffy particles. These particles are likely Pb(II)-related precipitates. Additionally, the rich surface functional groups present on IALG-BC facilitate further Pb(II) adsorption through ion exchange, coordination, and other interactions (see Equations (19)–(23)) [35,36].

(17) $${\mathrm{Na}}_{2}{\mathrm{CO}}_{3}+{\mathrm{Pb}}^{2+}\to {\mathrm{PbCO}}_{3}↓+{\mathrm{Na}}^{+}$$

(18) $${\mathrm{Na}}_{2}{\mathrm{SiO}}_{3}+{\mathrm{Pb}}^{2+}\to {\mathrm{PbSiO}}_{3}↓+{\mathrm{Na}}^{+}$$

(19) $${\mathrm{Biochar}}_{-\mathrm{COOH}}^{-\mathrm{COOH}}+{\mathrm{Pb}}^{2+}\to {\mathrm{Biochar}}_{-\mathrm{COO}}^{-\mathrm{COO}}\mathrm{Pb}+2H^{+}$$

(20) $${\mathrm{Biochar}}_{-\mathrm{COONa}}^{-\mathrm{COONa}}+{\mathrm{Pb}}^{2+}\to {\mathrm{Biochar}}_{-\mathrm{COO}}^{-\mathrm{COO}}\mathrm{Pb}+2{\mathrm{Na}}^{+}$$

(21) $${\mathrm{Biochar}}_{-\mathrm{OH}}^{-\mathrm{OH}}+{\mathrm{Pb}}^{2+}\to {\mathrm{Biochar}}_{-O}^{-O}\mathrm{Pb}+2H^{+}$$

(22) $${\mathrm{Biochar}}_{-\mathrm{ONa}}^{-\mathrm{ONa}}+{\mathrm{Pb}}^{2+}\to {\mathrm{Biochar}}_{-O}^{-O}\mathrm{Pb}+2{\mathrm{Na}}^{+}$$

(23) $$\mathrm{Biochar}-\ddot{C=C}+{\mathrm{Pb}}^{2+}\to \mathrm{Biochar}-C=C:{\mathrm{Pb}}^{2+}$$

#### 2.4.3. Thermodynamic Analysis

The thermodynamic parameters of the adsorption process, including the Gibbs free energy (ΔG^0^), enthalpy (ΔH^0^), and entropy (ΔS^0^), are instrumental in revealing the underlying mechanisms of the reaction. These parameters can be determined using Equations (24)–(28):

(24) $$∆G^{0}=-RT\ln K_{e}$$

(25) $$K_{w}=\frac{Q_{e}}{C_{e}}$$

(26) $$K_{e}=\rho K_{w}=\rho \frac{Q_{e}}{C_{e}}$$

(27) $$∆G^{0}=∆H^{0}-T∆S^{0}$$

(28) $$\ln K_{e}=-\frac{∆H^{0}}{RT}+\frac{∆S^{0}}{R}$$

In these equations, *K*_e_ represents the dimensionless adsorption equilibrium constant; *R* is the universal gas constant (8.314 J/(mol·K)); *T* denotes the absolute temperature (K); *Q*_e_ is the equilibrium adsorption capacity (mg/g); *C*_e_ is the equilibrium concentration of the adsorbate after adsorption (mg/L); and *ρ* is the density of water (g/cm^3^).

The thermodynamic parameters for the adsorption of Pb(II) by IALG-BC and A-IALG-BC, as shown in Table 6, provide critical insights into the underlying adsorption mechanisms. At different ambient temperatures, the obtained Δ*G*^0^ values are consistently negative, indicating that the adsorption of Pb(II) by both IALG-BC and A-IALG-BC is a spontaneous process. Moreover, with increasing temperature, the Δ*G*^0^ values show a decreasing trend, suggesting that higher temperatures enhance the spontaneity of the Pb(II) adsorption by IALG-BC and A-IALG-BC. This observation aligns with findings reported by Wang et al. regarding the adsorption of heavy metal ions by biochar [49]. The positive Δ*H*^0^ values across all conditions further indicate that the adsorption of Pb(II) by IALG-BC and A-IALG-BC is an endothermic process, signifying that elevated temperatures favor the adsorption process, corroborating the analysis shown in Figure 8. Additionally, the Δ*S*^0^ values, which represent the degree of disorder during the reaction, indicate that the adsorption of Pb(II) by IALG-BC involves a significantly higher degree of disorder compared to that by A-IALG-BC.

## 3. Materials and Methods

### 3.1. Raw Materials and Reagents

IALG, lead chloride (PbCl_2_), sodium hydroxide (NaOH), and hydrogen chloride (HCl) were supplied by Sigma-Aldrich. Deionized water, generated via a Milli-Q water purification system (Millipore, Inc., Bedford, MA, USA), was utilized for the preparation of all solutions. To regulate the pH of the solution, adjustments were made using 0.1 M NaOH and HCl. The ash content of IALG is 20.16 wt.%, with sodium being the main component (14,059 mmol/kg). The ash in IALG was treated using a mixture of nitric acid and hydrofluoric acid. After multiple washes with deionized water, it was dried to obtain ash-free lignin (A-IALG).

### 3.2. Preparation of Biochar

A sample of 50 g of IALG was weighed and placed in a lidded ceramic crucible, which was then introduced into a muffle furnace. The pyrolysis process was conducted at a temperature of 700 °C for a duration of 2 h. Upon completion of the pyrolysis, the muffle furnace was allowed to cool naturally to room temperature. The resulting solid residue was subsequently removed and ground to produce the target biochar, designated as IALG-BC. Following the same procedure, A-IALG-BC was also prepared.

### 3.3. Characterization of Samples

An amount of 10 g of IALG was precisely measured using a ceramic crucible and positioned within a muffle furnace. The sample was subjected to calcination at 600 °C for a duration of 4 h. Upon completion, the mass of the residual solid was determined to calculate the ash content of the IALG. Subsequently, the IALG was digested with aqua regia and inductively coupled plasma optical emission spectrometry was employed (720, Agilent, Inc., Palo Alto, CA, USA) to quantify the Na concentration in the resultant solution, thereby ascertaining the sodium content within the IALG. Approximately 5 mg of the sample was weighed and placed into an alumina crucible. The sample was subjected to a controlled temperature increase from ambient room temperature to 800 °C, with a heating rate of 10 °C/min. The changes in the sample’s mass as a function of the reaction temperature were analyzed using a thermogravimetric analyzer (TG209 F3, Netzsch, Inc., Bavaria Free State, Germany). The specific surface area and pore structure characteristics of the sample were determined using a nitrogen adsorption/desorption apparatus (Nova 2200e, Quantachrome Instruments, Boynton Beach, FL, USA). The specific surface area of the sample was measured using the BET method, utilizing adsorption data within the relative pressure range of 0.01 to 0.2 (P/P_0_). The total pore volume was derived from the amount of nitrogen adsorbed at a relative pressure of approximately 0.99 (P/P_0_). The mesopore specific surface area and mesopore volume were obtained by calculating the differences from the micropore surface area and micropore volume, which were determined through t-plot analysis. Functional groups present on the sample surface were characterized using Fourier transform infrared spectroscopy (FT-IR, Nicolet 6700, Thermo Fisher Scientific, Inc., Waltham, MA, USA). The microstructure of the sample was primarily examined via scanning electron microscopy at an acceleration voltage of 20 kV (SEM, Inspect F50, FEI, Thermo Fisher Scientific, Inc., Waltham, MA, USA). The crystalline phases present in the sample were identified using an X-ray diffractometer under the conditions of nickel-filtered Cu Kα radiation (λ = 0.15406 nm) at a current of 20 mA and a voltage of 30 kV (XRD, SmartLAB 3, Rigaku Corporation, Saitama Prefecture, Japan).

### 3.4. Pb(II) Adsorption

A quantity of 0.1 g of biochar was added to 50 mL of Pb(II) solution in a wide-mouth Erlenmeyer flask. The suspension was placed in a constant temperature incubator and agitated at a speed of 120 r/min. Upon completion of the reaction, the mixture was separated using a 0.45 µm filter membrane. The concentration of Pb(II) in the filtrate was determined using an atomic absorption spectrometer (FAAS-M6, Thermo Fisher Scientific, Inc., Waltham, MA, USA). The initial concentration of Pb(II), reaction time, reaction temperature, and solution pH were set to 50–1000 mg/L, 2–8 h, 15–45 °C, and 1–5, respectively. Each sample was set up in triplicate, and the data used are the average of the three sets of values.

## 4. Conclusions

The high ash content in IALG acted as an efficient catalyst, significantly reducing the activation energy required during the primary pyrolysis stage to 14.27 J/mol, thereby enhancing the development of the resultant biochar’s porous structure. Moreover, the abundant minerals in IALG facilitated the deoxygenation and dehydrogenation processes within the carbon materials, which resulted in the attenuation or disappearance of the characteristic peaks associated with the functional groups. Following pyrolysis, the Na-related minerals were transformed into particles ranging from 80 to 150 nm in size, including Na_2_SiO_3_, Na_2_CO_3_, and NaCl. IALG-BC demonstrated a markedly superior Pb(II) adsorption performance relative to that of A-IALG-BC. According to the Langmuir model, the theoretical maximum adsorption capacity of IALG-BC for Pb(II) was 809.09 mg/g, which was approximately 40 times that of A-IALG-BC. The Na-related minerals present in IALG-BC functioned as active adsorption sites, playing an essential role in the efficient adsorption of Pb(II) through precipitation. This study elucidates the potential of industrial alkali lignin as a cost-effective and efficient adsorbent for the remediation of wastewater containing heavy metals. Nonetheless, further research is warranted to explore the development of recovery methods for the spent adsorbents and to establish effective regeneration processes.

## Figures and Tables

**Table 1:** Kinetic parameters of IALG and A-IALG.

	*E* (J/mol)	*A* (1/min)	*R* ^2^
IALG	14.27	5.78	0.9982
A-IALG	38.44	10.39	0.9896

**Table 2:** Porosity properties of IALG-BC and A-IALG-BC.

	Specific Surface Area (m^2^/g)	Mesopore Specific Surface Area (m^2^/g)	Total Pore Volume (cm^3^/g)	Mesopore Pore Volume (cm^3^/g)
IALG-BC	106.65	38.09	0.09	0.02
A-IALG-BC	2.81	0.91	0.01	/

**Table 3:** Adsorption isotherm fitting parameters for the adsorption of Pb(II) on IALG-BC and A-IALG-BC.

	Langmuir Model			Freundlich Model		
	*Q*_m_ (mg/g)	*K*_l_ (L/mg)	*R* ^2^	*K*_f_ (mg^(1−n)^L^n^/g)	*n*	*R* ^2^
IALG-BC	809.09	0.005	0.997	12.69	1.50	0.961
A-IALG-BC	20.85	0.003	0.995	0.66	2.12	0.965

**Table 4:** Comparison of the theoretical maximum adsorption capacity of Pb(II) according to Langmuir model fitting by various biochars.

No.	Biochars	Adsorption Conditions	Theoretical Maximum Adsorption Capacity (mg/g)	Ref.
1	From peanut shells via pyrolysis at 300 °C for 2 h.	Initial concentration in the range of 50–2000 mg/L at 25 °C for 3 h.	210.10	[18]
2	From pomelo fruit peels via pyrolysis at 500 °C for 45 min.	At 30 °C with solution pH of 5 for 2 h.	92.13	[43]
3	From cotton straw via pyrolysis at 300 °C for 2 h.	Initial concentration in the range of 5–300 mg/L at 25 °C with solution pH of 5.5 for 12 h.	102.70	[44]
4	From corn stalks via pyrolysis at 300 °C for 1 h.	Initial concentration in the range of 80–500 mg/L at 30 °C with solution pH of 5 for 8 h.	15.66	[45]
5	From corn stalks via pyrolysis at 500 °C for 2 h.	Initial concentration in the range of 100–1000 mg/L at 25 °C with solution pH of 5 for 4 h.	40.98	[39]
6	From sewage sludge via pyrolysis at 700 °C for 1 h.	Initial concentration in the range of 5–300 mg/L at room temperature for 24 h.	7.56	[46]
7	From industrial alkali lignin via pyrolysis at 700 °C for 2 h (IALG-BC).	Initial concentration in the range of 50–1000 mg/L at 25 °C with solution pH of 5 for 4 h.	809.09	This work

**Table 5:** Adsorption kinetic fitting parameters for the adsorption of Pb(II) on IALG-BC and A-IALG-BC.

	*q*_e_	Pseudo-First-Order Model			Pseudo-Second-Order Model		
		*k*_1_ (1/h)	*Q*_e_ (mg/g)	*R* ^2^	*k*_2_ (g/(mgh))	*Q*_e_ (mg/g)	*R* ^2^
IALG-BC	92.82	7.08	89.97	0.906	0.15	92.96	0.991
A-IALG-BC	12.95	0.77	10.69	0.924	0.06	13.03	0.989

**Table 6:** Thermodynamic parameters for the adsorption of Pb(II) on IALG-BC and A-IALG-BC.

	Δ*G*^0^ (kJ/mol)				Δ*H*^0^	Δ*S*^0^
	288 K	298 K	308 K	318 K	kJ/mol	J/(mol K)
IALG-BC	−12.77	−15.82	−17.00	−20.81	59.75	251.99
A-IALG-BC	−2.11	−3.97	−4.80	−6.08	34.56	128.06

## Data Availability

Data will be made available upon request.
